# Serotonergic Mechanisms of Oocyte Germinal Vesicle Breakdown in the Mud Crab, *Scylla paramamosain*

**DOI:** 10.3389/fphys.2019.00797

**Published:** 2019-06-19

**Authors:** Yanan Yang, Dongdong Lin, Chenchang Bao, Huiyang Huang, Haihui Ye

**Affiliations:** ^1^School of Marine Sciences, Ningbo University, Ningbo, China; ^2^College of Ocean and Earth Sciences, Xiamen University, Xiamen, China

**Keywords:** serotonin (5-HT), oocyte maturation, germinal vesicle breakdown (GVBD), cAMP, mud crab

## Abstract

The mechanism of serotonin (5-HT)-induced oocyte germinal vesicle breakdown (GVBD) in the mud crab, *Scylla paramamosain*, was investigated in this study. Histological staining showed that there were two meiotic arrests in oocyte, appearing at prophase I and metaphase I. This result indicated that meiosis I arrest at prophase I in *S. paramamosain* was similar to that of vertebrates, but meiosis II arrest at metaphase I was different from that of vertebrates. Resumption of oocytes arrest at meiosis prophase I could be induced by 5-HT rapidly within 5 min in *S. paramamosain*. We obtained the sequence of the 5-HT receptor type 1A (*5-HTR_1A_*) from the NCBI database, and found that *5-HTR_1A_* was expressed in oocytes and follicle cells. In addition, we found that an agonist 8-OH-DPAT which binds *5-HTR_1A_* induced GVBD and an antagonist WAY100635 which inhibited 5-HT induced GVBD in *S. paramamosain*. This result showed that *5-HTR_1A_* mediated the regulation of oocyte GVBD by 5-HT. To explore the functional mechanism of 5-HT in inducing oocyte GVBD, forskolin, a cAMP agonist was used. Results showed that, forskolin significantly blocked 5-HT-induced GVBD, and there was a negative correlation between GVBD rate and cAMP level. Our data indicate that there are two meiotic arrests in *S. paramamosain*, and the resumption of prophase I arrest can be induced by 5-HT, which binds to *5-HTR_1A_*, and this process is mediated by cAMP, which acts as negative regulator via cAMP signaling pathway.

## Introduction

During oogenesis, animal oocytes are usually arrested at meiosisprophase I and their nucleus is enlarged in a process known as germinal vesicle (GV). Meiosis is resumed in response to a stimulus and the first sign of meiosis initiation is the germinal vesicle breakdown (GVBD) (Eppig, 1982). The oocytes then undergo metaphase I or II before fertilization (Masui and Clarke, 1979; Sagata, 1997), which are specific in different species. In contrast, fertilization takes place at prophase I in only a few species, after which oocytes complete meiosis through GVBD, such as clam *Spisula* and *Mactra* (Longo, 1983; Fong et al., 1996). Resumption of oocytes arresting at meiosis prophase I is induced by external stimuli such as hormones, neurotransmitters and other molecules (Hirai et al., 1988; Guerrier et al., 1993; Mita, 2000; Nebreda and Ferby, 2000). For example, oocytes maturation is thought to be triggered by progesterone and insulin in amphibian (Nebreda and Ferby, 2000), by serotonin in many bivalves (Hirai et al., 1988; Guerrier et al., 1993), and by 1-Methyladenine in starfish (Mita, 2000).

Serotonin (5-hydroxytryptamine, 5-HT) is a biogenic amine neurotransmitter and is widely used in animal phyla. It modulates diverse complex behaviors, including sexual (Hull et al., 2004), feeding behavior (Higgins et al., 1992), and sleep-wake-arousal cycle (Jacobs and Azmitia, 1992) in vertebrates. In invertebrates, 5-HT regulates various functions such as circadian rhythmicity (Eskin and Takahashi, 1983), escape behaviors (Bicker and Menzel, 1989), and ovarian maturation (Meeratana et al., 2006). Previous studies in crustaceans revealed that, 5-HT induces ovarian maturation in giant freshwater prawn *Macrobrachium rosenbergii*, in mud crab *Scylla olivacea* and other crustacean species (Vaca and Alfaro, 2000; Meeratana et al., 2006; Wongprasert et al., 2006; Kornthong et al., 2013). 5-HT induced reproduction is mediated either by release of gonad stimulating hormone or by stimulating the release of red pigment concentration hormone in many crustacean species (Fingerman, 1997; Nagaraju, 2011; Swetha et al., 2011). In addition, 5-HT has been reported to stimulate oocyte maturation in some marine nemertean worms (Stricker and Smythe, 2000, 2001) and mollusks (Hirai et al., 1988; Guerrier et al., 1993). In the mechanism of 5-HT-induced GVBD in oocytes of marine nemertean worms, high concentration of cAMP is required to activate the maturation promoting factor (MPF), and this is also the case in other species, including brittle stars and cnidarians (Freeman and Ridgway, 1988; Yamashita, 1988; Deguchi et al., 2011). In contrast, maturing oocytes of the starfish requires low levels of cAMP, as well as in the frog (Taieb et al., 1997; Ferrell, 1999). Being a trigger of GVBD, 5-HT acts via receptors to mediate oocytes maturation (Krantic et al., 1993; Ram, 1994). A study on mollusks illustrated that the GVBD in zebra mussel *Dreissena polymorpha* can be mediated by 5-HT receptors (Ram, 1994). Similarly, in the surf clam *Spisula solidissima*, GVBD was found to be mediated by 5-HT membrane receptors which are different from those in *Drosophila* (Krantic et al., 1993).

Compared with invertebrate organisms, few studies have investigated the oocyte maturation in crustacean species. In previous studies, it was reported that Cdc2 kinase and cyclin B are highly expressed in GVBD oocytes, and microRNA miR-2 and miR-133 can regulate oocyte meiosis by inhibiting the translation of cyclin B in Chinese mitten crab, *Eriocheir sinensis* (Qiu and Liu, 2009; Feng and Qiu, 2011; Song et al., 2014). We recently reported that GVBD is inhibited by short neuropeptide F (sNPF) and cAMP level is reduced during GVBD process in the mud crab, *S. paramamosain* (Bao et al., 2018). However, the mechanisms of hormonally induced oocyte GVBD in crustacean are not well-understood. In this study, we investigated the resumption of oocyte arrests at prophase I of meiosis due to 5-HT, and explore the mechanism of 5-HT-induced GVBD in *S. paramamosain*.

## Materials and Methods

### Ethics Statement

This study was carried out in accordance with the recommendations of the Institutional Animal Care and Use Committee (IACUC) of the Xiamen University. The protocol was approved by the Committee on the Ethics of Animal Experiments of the Xiamen University. Adult female crabs *S. paramamosain* (300–350 *g*, with a carapace width of 11.5–12.0 cm) were obtained from the local aquatic market in Xiamen, Fujian Province, China. Crabs were reared in seawater tanks with at 27 ± 1°C (salinity 29 ± 1 ppt) and fed on live clam *Ruditapes philippinarum* for a week before experiments. All crabs used in experiments were under normal physiological conditions without any induction or treatment. For ovary harvesting, crabs were placed on ice anesthetization for 15 min.

### Separation of Oocytes and Follicle Cells

Ovaries at late vitellogenic stage (stage IV) were used to collect serum and used to separate oocytes and follicle cells. Characteristics of late vitellogenic stage are: oocytes peel off from ovary easily; diameter of the oocyte in this stage is approximately 240 μm; cytoplasm is occupied with massive yolk globules and vitellogenesis is almost complete; the nuclear membrane is clear and complete, and chromosome can hardly be observed (Shangguan et al., 1991; Islam et al., 2010).

Haemolymph was withdrawn from the arthrodial membrane of last walking leg using syringe, and stored at -20°C before used. The haemolymph was unfreeze at 4°C, and serum was collected for oocyte separation. Oocytes and follicle cells were separated as previously described in mud crab (Yang et al., 2018). Ovaries at late vitellogenic stage were dissected from crabs, and then placed in a glass culture dish with icy serum (4°C). The follicles layer was grabbed with fine forceps and shook carefully under a microscope. The scattered oocytes released into the dish were collected by filtering with a strainer. The separated follicle layers and denuded oocytes were sampled and used for GVBD observation and RNA extraction.

### GVBD Observation

Fully grown oocytes showing a clear GV were selected to examine GV and GVBD in this study. After seperation from the ovary, oocytes were incubated in 6-well plate with 100% crab serum at room temperature (26 ± 1°C), and then some oocytes underwent GVBD spontaneously within 30 min after the isolation. GV and GVBD were determined by placing oocytes in a clearing solution (formaldehyde, ethanol, acetic acid, 30:40:1) followed by microscopic examination. The ovarian stages used in this study were late vitellogenesis stage (stage IV) and mature stage (stage V). Characteristics of mature stage are: oocytes peel off from ovary easily; diameter of the oocyte is approximately 260 μm; large yolk globules in the entire cytoplasm; some nucleus concentrated, or arrested at metaphase I of meiosis (Shangguan et al., 1991; Chen et al., 2004).

### Histological Staining

Germinal vesicle stage, GVBD stage and metaphase I oocytes were fixed in Bouin’s fixative for 12 h at 4°C, dehydrated, and then embedded in paraffin. Sections of 7 μm-thickness were deparaffinized, rehydrated and then stained with hematoxylin and eosin. Images were taken by Olympus BX51 microscope (Olympus, Tokyo, Japan) equipped with an UPlanFL 20×/0.5 numeric aperture and UPlanFL 40×/0.75 numeric aperture objective lens. Images were captured by Olympus DP71 digital camera system and processed by Image-Pro Plus software.

### RNA Extraction and cDNA Synthesis

Intact ovary, denuded oocytes and follicle cells were excised from the crab and immediately flash-frozen in liquid nitrogen. Total RNA was extracted using TRIzol RNA isolation reagent (Invitrogen, Carlsbad, CA, United States) according to the manufacturer’s instructions and the total RNA concentration, quality and integrity were determined by a NanoDrop 2000 spectrophotometer (Thermo Fisher Scientific, Pittsburgh, PA, United States). Genomic DNA was removed by digestion with DNase I (Thermo Fisher Scientific, Vilnius, Lithuania) and the first strand cDNA was synthesized from 1 μg of total RNA by the Revert Aid First Strand cDNA Synthesis Kit (Thermo Fisher Scientific, Vilnius, Lithuania) according to the manufacturer’s instructions.

### Determination of Expression Pattern of *5-HTR_1A_* in Ovary by Semiquantitative PCR

The semiquantitative PCR was performed to detect the expression level of 5-HT receptor type 1A (*5-HTR_1A_*) in the intact ovary, denuded oocytes and follicle layer. The fragment of *5-HTR_1A_* cDNA sequence was obtained from transcriptome data, and was verified by DNA sequencing. The following primer pair consistently amplified a 167 bp fragment of *5-HTR_1A_* were used for PCR. Primers were designed according to the cDNA sequences (GenBank: MK645884) primer SF1 (GGTGACGCAGGTGGACTACATA) and SR1 (GGGACACCAGGCACTTCTTAT). Housekeeping gene *β*-actin (GenBank: GU992421) with primers F8 (GAGCGAGAA ATCGTTCGTGAC) and R9 (GGAAGGAAGGCTGGAA GAGAG) was used as reference gene, and target genes amplified using water as the template were used as blank control. The reaction system was prepared with a volume of 25 μL containing 12.5 μL of 2 × Premix Ex TaqII (Takara Bio), 2 μL cDNA, 1 μM each primer and 8.5 μL H_2_O. The PCR cycling procedure was as follows: an initial denaturing at 94°C for 3 min, 38–40 cycles at 94°C for 30 s, 55°C for 30 s and 72°C for 30 s, followed by an extension at 72°C for 10 min. The products were assessed by 1.5% agarose gel electrophoresis. A semiquantitative PCR was repeated three times for each sample.

### Regulation of Oocyte Maturation by 5-HT

To investigate whether GVBD can be induced by 5-HT *in vitro*, oocytes were exposed to 10^–5^ M 5-HT for 0, 5, 15, 30, and 60 min at room temperature (26 ± 1°C). The concentration of 5-HT was refered to the previous studies in mollusca (Deguchi and Osanai, 1995; Moreau et al., 1996; Fong, 1998). As a *5-HTR_1A_* agonist, 10^–5^ M 8-hydroxydipropylaminotetralin hydrobromide (8-OH-DPAT) was added to the fully grown oocytes showing a clear GV. After 5 and 15 min, oocytes were scored in terms of GVBD occurrence. In a similar experiment, oocytes were preincubated with *5-HTR_1A_* antagonists 25 nM WAY100635 for 10 min, followed by incubation with 5-HT for 5 and 15 min. Spontaneous maturation of untreated GV oocytes were used as the control group. About 100–120 oocytes were counted in each group. GVBD rates were defined as the percentage of oocytes that completed GVBD. All the experiments were repeated four times.

### Influence of 5-HT and Forskolin Co-treatment on Oocyte Maturation

Germinal vesicle breakdown rates were determined as described above. Briefly, GVBD rates were recorded at 0, 5, 15, 30, and 60 min for 5-HT (10^–5^ M)-treated oocytes as well as 5-HT (10^–5^ M) and forskolin (10^–4^ M) (three replicates per treatment).

### Measurement of cAMP Levels

The measurement of cAMP level was performed at 0, 5, and 15 min. Oocytes were lysed and the concentration of cAMP was determined using a competitive binding technique based on enzyme-linked immunosorbent assay (ELISA) (cAMP EIA kit, Cayman) according to the manufacturer’s instructions.

### Statistical Analysis

All values are expressed as mean ± SD. Statistical analysis was performed using SPSS 22.0. After the homogeneity tests, One-way ANOVA followed by Duncan’s test was used to determine the significant differences in GVBD rates or cAMP levels. Significances were accepted at *P* < 0.05.

## Results

### GVBD Examination

**FIGURE 1:**
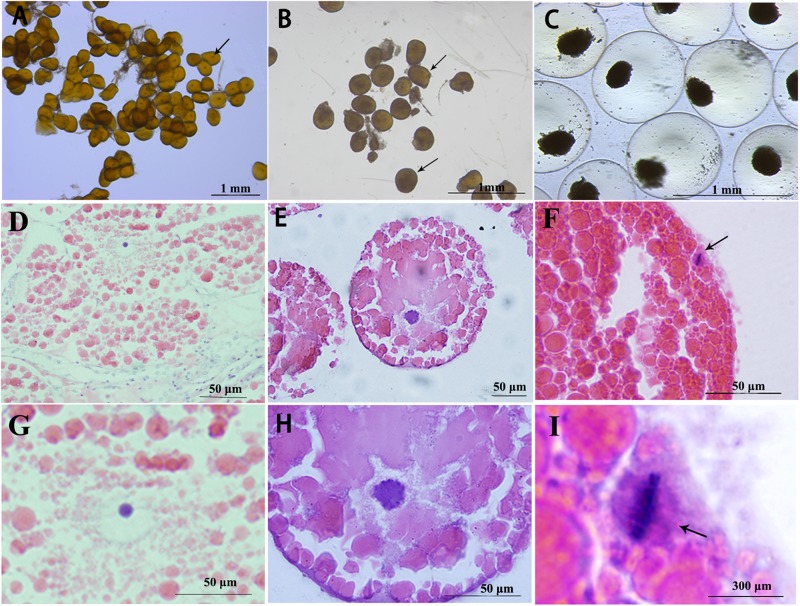
Characteristics of three stages oocytes, visualized under various magnifications. GV stage **(A,D,G)**, arrows indicate GV stage oocytes; GVBD stage **(B,E,H)**, arrows indicate GVBD stage oocytes; and metaphase I oocytes **(C,F,I)**, arrows indicate chromosomes arranged at the equator.

The arrest of fully grown oocytes of *S. paramamosain* at meiosis prophase I was identified by the presence of a large nucleus known as GV, which is the first meiotic arrest (Figures 1A,D,G). Meiosis resumes and the sign of meiosis progression is GVBD (Figures 1B,E,H). Oocytes are arrested again at metaphase I of meiosis and this is accompanied by arrangement of compact chromosomes at the equator of the spindle (Figures 1C,F,I). This is the second meiotic arrest in oocytes of *S. paramamosain*.

### 5-HT-Induced Oocyte Maturation

**FIGURE 2:**
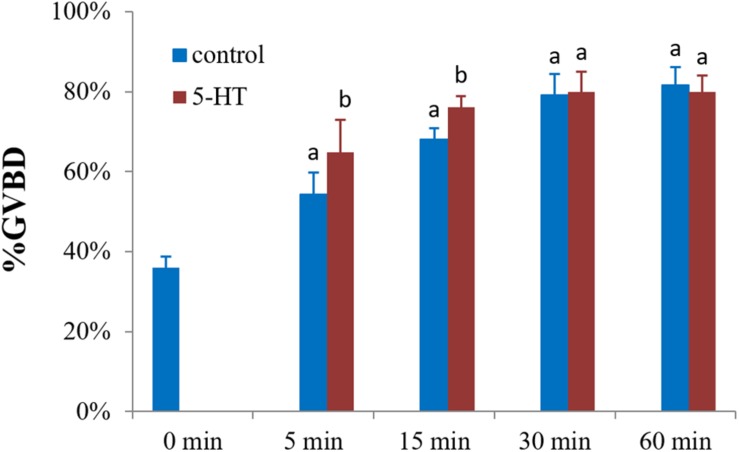
Effects of 5-HT on GVBD rate of oocyte in *S. paramamosain.* Oocytes were exposed to 5-HT for 0, 5, 15, 30, and 60 min at room temperature (26 ± 1°C), oocytes were scored in terms of GVBD occurrence. The GVBD rates were defined as the percentage of oocytes that completed GVBD. All experiments were repeated four times.

To explore the potential role of 5-HT in oocyte maturation, denuded oocytes (GV stage) were evaluated. The 5-HT induced resumption of meiosis in *S. paramamosain*. In comparison to the control group, GVBD rates of oocytes were increased by 10 and 8% (*P* < 0.05) after incubating oocytes with 5-HT for 5 and 15 min (Figure 2). However, no significant difference was observed between the 5-HT treated and the control group at 30 and 60 min. Interestingly, meiosis was rapidly reinitiated, and the rate of spontaneous maturation reached at 36% at 0 min without any treatment (Figure 2).

### Characteristics of *5-HTR_1A_*

**FIGURE 3:**
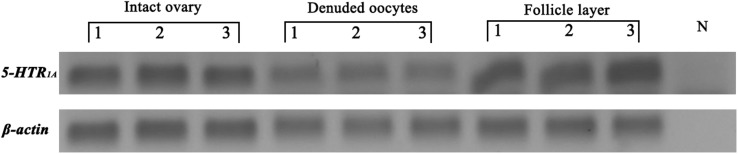
Localization of *5-HTR_1A_* in the intact ovary, denuded oocytes, and follicle layer, respectively. The reference gene (*β*-actin) was expressed in each compartment. N indicates the blank control.

Semi quantitative PCR was performed to analyze the expression of *5-HTR_1A_* in oocytes and follicle cells from ovaries at late vitellogenic stage. The results showed that *5-HTR_1A_* were expressed in oocytes and follicle cells (Figure 3). *β*-actin (used as the reference gene) was present in all templates, but no signals were observed in the blank control (using distilled water as the template) (Figure 3).

**FIGURE 4:**
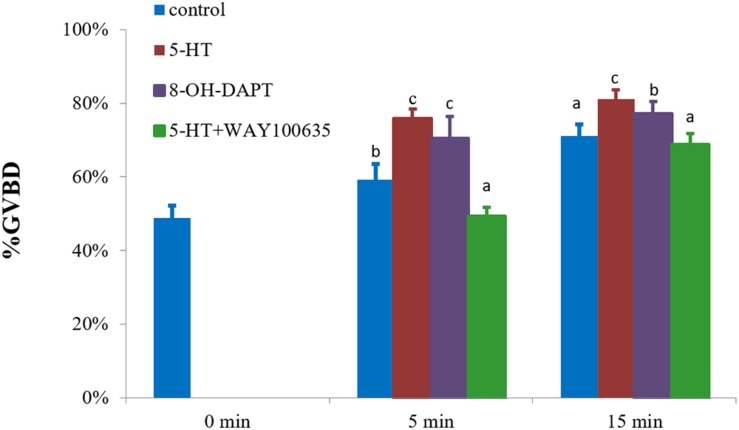
Effects of 8-OH-DPAT and WAY100635 on the GVBD rate of oocyte in *S. paramamosain.* Oocytes were exposed to 8-OH-DPAT and WAY100635 followed by 5-HT for 0, 5, and 15 min at room temperature (26 ± 1°C), oocytes were scored in terms of GVBD occurrence. The GVBD rates were defined as the percentage of oocytes that completed GVBD. All experiments were repeated four times.

The characteristic of *5-HTR_1A_* was examined using a specific agonist 8-OH-DPAT. It was found that 8-OH-DPAT effectively induced GVBD at 5 and 15 min with GVBD rates of 70 and 77% (*P* < 0.05), respectively (Figure 4). After 15 min of treatment, 5-HT-treated group (positive control) showed a stronger induction effect than 8-OH-DPAT-treated group (Figure 4).

The inhibitory effect of the specific antagonist WAY100635 was also examined. The rate of GVBD after WAY100635 pretreatment and then exposure to 5-HT was 49%, which was significantly lower than that of 5-HT-treated group with 76% (positive control) and control group with 59% (*P* < 0.05, Figure 4) at 5 min. A similar result was observed at 15 min, in which the rate of GVBD in WAY100635 pretreatment followed by 5-HT was 69%, which was significantly lower than that of 5-HT-treated group with 81%, but it was not different from the rates of the control group (Figure 4).

### Influence of cAMP Signaling Pathway in Oocyte Maturation

**FIGURE 5:**
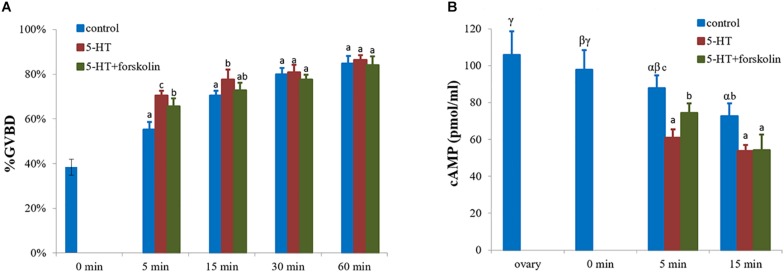
Effects of forskolin on the GVBD rate and cAMP levels of oocytes in *S. paramamosain.* Oocytes were exposed to 5-HT and forskolin followed by 5-HT for 0, 5, 15, 30, and 60 min at room temperature (26 ± 1°C), oocytes were scored in terms of GVBD occurrence **(A)**. The GVBD rates were defined as the percentage of oocytes that completed GVBD. cAMP levels were tested in each group at 0, 5, and 15 min **(B)**. All experiments were repeated four times.

As an adenylate cyclase activator, forskolin inhibited GVBD process which was induced by 5-HT (Figure 5A). The rate of GVBD in forskolin-pretreated cells followed by 5-HT was 66%, which was significantly lower than that of 5-HT-treated group with 70% (positive control), but higher than the control group with 55% (*P* < 0.05, Figure 5A) at 5 min. Yet, the rate of GVBD in the experimental group was still lower than that of 5-HT-treated group by 73% (*P* < 0.05) at 15 min. There was no significant difference among the three groups at 30 and 60 min (Figure 5A).

cAMP levels were measured at 0, 5, and 15 min. The highest cAMP level of 97.8 pmol/ml was observed in the control group at 0 min, with a GVBD rate of 38%. Subsequently, the GVBD rate increased with time but the levels of cAMP decreased (Figure 5). At 5 min, the levels of cAMP in 5-HT treated group, forskolin-pre-treatment with 5-HT group, and control group were different for each other (*P* < 0.05, Figure 5B). As shown in Figure 5, the highest and lowest GVBD rates were observed in 5-HT-treated group and control group, respectively. In contrast, the highest and lowest cAMP levels were observed in control group and 5-HT treated group, respectively. At 15 min, the profiles of cAMP levels were similar to those at 5 min (Figure 5B).

## Discussion

In *S. paramamosain*, mature oocyte are arrested at metaphase I of meiosis until fertilization (Chen et al., 2004). Histological staining results showed that there were two meiotic arrests in *S. paramamosain*, the second one of which is metaphase I of meiosis, (Figures 1C,F,I), while the first one occurs at prophase I of meiosis (Figures 1A,D,G). These findings are similar to those reported in previous studies in penaeid shrimp (Anderson et al., 1984; Yano, 1988). Unlike the mud crab, some species only have once meiotic arrest, such as clam *Spisula* and *Mactra* (Longo, 1983; Fong et al., 1996). In vertebrate, fully grown oocytes are arrested at prophase I of meiosis, resumes in response to stimuli, and then the oocytes are arrested at metaphase II of meiosis, waiting for fertilization. Our results indicated that meiosis I arrest at prophase I in *S. paramamosain* was similar to that of vertebrates, but meiosis II arrest at metaphase I was different from that of vertebrates (Schmitt and Nebteda, 2012). The distinct times and phases of meiotic arrests varies with species.

As a hormone, 5-HT rapidly induced oocyte maturation in mud crab within 5 min (Figure 2). Since 5-HT is found in bivalve ganglia and gonads, it has been used as an inducer of oocyte GVBD (Ram et al., 1992; Colas and Dubé, 1998). Previous studies in *S. paramamosain* indicated that 5-HT was expressed in cerebral ganglia and eyestalk, and that it can stimulate ovarian development *in vivo* (Ye et al., 2003; Huang et al., 2005a, 2005b). Surprisingly, spontaneous GVBD with a rate of up to 36% occurred after isolation of follicle cells from ovaries, which differed from a previous study on bivalves (Yi et al., 2002; Wang and Zhang, 2011). In mammals, accessory cells can facilitate meiotic block by transferring small molecules to oocytes through gap junctions (Eppig et al., 1983). In *S. paramamosain*, the high spontaneous GVBD rates can be explained by the decline in cAMP levels (which will be discussed below) and some paracrine factors in follicular cells, which inhibit GVBD, such as sNPF and BMP7 (Bao et al., 2018; Yang et al., 2018).

In this study, the sequence of *5-HTR_1A_* was obtained from NCBI database (GenBank: MK645884). Figure 3 shows that *5-HTR_1A_* was expressed in oocytes and follicle cells. This result is consistent with that of a previous report in surf clam (Krantic et al., 1993). Sequence analysis showed that *5-HTR_1A_* is a G protein-coupled receptor. Thus, we speculated that the receptor of *5-HTR_1A_* might mediate the effect of 5-HT on oocyte GVBD. Indeed, the results showed that 8-OH-DPAT, the agonist of *5-HTR_1A_*, induced GVBD rapidly (Figure 4). WAY100635 is the first potent, selective and silent antagonist of *5-HTR_1A_* which displays regional binding sites as 8-OH-DPAT in human and rat brains (Fletcher et al., 1995; Burnet et al., 1997). As reported in vertebrates, WAY100635 effectively inhibited 5-HT-induced GVBD in *S. paramamosain* (Figure 4). This result shows that *5-HTR_1A_* mediated the effect of 5-HT on oocyte GVBD. The 8-OH-DPAT was found to be an effective agonist of *5-HTR_1A_* receptor which plays a role in the regulation of oocyte GVBD in bivalves (Kadam and Koide, 1989; Fong et al., 1994, 1996). For example, application of 5-HT and 8-OH-DPAT induced GVBD in zebra mussel (*D. polymorpha*) oocytes (Fong et al., 1996). However, neither 5-HT nor 8-OH-DPAT induced GVBD in *Mactra chinensis* oocytes (Fong et al., 1996). The controversial effects of 5-HT and 8-OH-DPAT in different bivalves could be ascribed to inherent species-specific factors.

Many signaling pathways, such as the Mos/mitogen-activated protein kinase (MAPK) pathway (Kosako et al., 1994), the phosphoinositide-phospholipase C (PI-PLC) pathway (Lefèvre et al., 2007) and the cAMP second messenger pathway (Schmitt and Nebteda, 2012), are involved in oocytes maturation. It has been demonstrated that intracellular second messenger cAMP inhibits the resumption of prophase I arrest in many animal oocytes, including rat, frog, and starfishes (Meijer et al., 1989; Heikinheimo and Gibbons, 1998; Ferrell, 1999), but enhances the resumption of prophase I arrest in brittle stars and cnidarians (Freeman and Ridgway, 1988; Yamashita, 1988; Deguchi et al., 2011). Furthermore, high level of cAMP induced by forskolin blocked the 5-HT-induced GVBD in *Spisula* oocytes (Sato et al., 1985). To determine the functional mechanism of 5-HT-induced oocyte GVBD, forskolin was used. Results showed that forskolin significantly blocked GVBD induced by 5-HT (Figure 5A), and there was a negative correlation between GVBD rate and cAMP level (Figure 5B). Previous studies revealed that cAMP in the follicle cells play a fundamental role in maintaining meiotic arrest in prophase I through the gap junctions, and may prevent spontaneous maturation in premature oocytes (Downs et al., 1989; Richard, 2007). An essential modulating factor, MPF acting downstream of cAMP pathways induces GVBD (Kishimoto, 2003). Therefore, we concluded that high level of cAMP prevented GVBD of oocytes in *S. paramamosain* as in many species (Dekel, 1996; Heikinheimo and Gibbons, 1998; Ferrell, 1999). Collectively, these datasets appears to indicate that cAMP inhibited 5-HT-induced oocyte GVBD in *S. paramamosain*, and *5-HTR_1A_* mediated the effect of 5-HT. It will be interesting to study whether the process of GVBD is mediated by other signaling pathways, for example the MAPK pathway and the PI-PLC pathway.

From the perspective of oocyte GVBD in crustaceans, this study reveals the mechanism of 5-HT in inducing GVBD of oocytes in *S. paramamosain*. There are two meiotic arrests in the mud crab, and the resumption of prophase I arrest can be induced by 5-HT through its receptor *5-HTR_1A_*. Moreover, this process is negatively mediated by cAMP signaling.

## Data Availability

All datasets for this study are included in the manuscript and/or the supplementary files.

## Ethics Statement

This study was carried out in accordance with the recommendations of the Institutional Animal Care and Use Committee (IACUC) of the Xiamen University. The protocol was approved by the Committee on the Ethics of Animal Experiments of the Xiamen University.

## Author Contributions

YY and HY designed the work. YY, DL, and CB performed the experiments. YY, DL, CB, and HY analyzed and interpreted the data. YY drafted the work. HY and HH revised the work.

## Conflict of Interest Statement

The authors declare that the research was conducted in the absence of any commercial or financial relationships that could be construed as a potential conflict of interest.
